# Medically Managed Locally Acquired Pulmonary Cystic Echinococcosis With Bacterial Superinfection in Northern Canada: A Case Report

**DOI:** 10.1155/crdi/9851244

**Published:** 2025-09-22

**Authors:** Ahmad F. Alenezi, Mohammed Redha, Cedric P. Yansouni, Sapha Barkati

**Affiliations:** ^1^Department of Medicine, McGill University, Montreal, Quebec, Canada; ^2^Department of Medicine, Division of Infectious Diseases, McGill University Health Centre, Montreal, Quebec, Canada; ^3^J.D. MacLean Centre for Tropical & Geographic Medicine at McGill University, Montreal, Quebec, Canada; ^4^Research Institute of the McGill University Health Centre, Montreal, Quebec, Canada

**Keywords:** albendazole, antiparasitic therapy, *Echinococcus granulosus*, hydatid cyst, lung cyst, surgical resection

## Abstract

**Background:** Primary pulmonary cystic echinococcosis (CE) is a zoonotic disease often caused by *Echinococcus granulosus* sensu lato complex. Although rare in North America, it can present significant diagnostic and therapeutic challenges.

**Case Presentation:** We report a 36-year-old male from Quebec, Canada, with locally acquired primary pulmonary CE who presented to the emergency department with a two-month history of shortness of breath, cough, and hemoptysis. Laboratory investigations showed mild leukocytosis and high eosinophil counts. A chest computed tomography (CT) scan revealed extensive multifocal consolidation in the right upper lobe (RUL) with a large 6-cm thick-walled cavity. *Echinococcus* serology was positive. Treatment was initiated with albendazole and praziquantel as well as antibiotics for pulmonary CE, with likely ruptured cyst and bronchoalveolar spillage complicated by a superimposed bacterial infection of the RUL. Follow-up imaging showed a decrease in the size of the cavitary lesion and regression of adjacent consolidations.

**Discussion:** Diagnosing and managing pulmonary CE is complex, as clinical presentations vary and imaging and serological tests have limitations. Treatment depends on factors such as cyst size, rupture status, and infection, with surgery as the main approach for viable cysts and albendazole used for ruptured cysts.

**Conclusion:** Pulmonary CE requires individualized management due to its varied presentations, with imaging and serology playing key but limited roles in the diagnosis. Medical management and monitoring were effective, while surgery was reserved for complex cases, with long-term follow-up and family screening essential for detecting recurrence and asymptomatic cases.

## 1. Background

Cystic echinococcosis (CE) is a zoonotic disease caused by cestodes of the genus *Echinococcus*, primarily affecting the liver [1]. The lungs are the second most commonly affected site [1]. Patients with CE may remain asymptomatic until complications arise [2]. Clinical manifestations of pulmonary CE can result from the mass effect due to the cyst's size or from complications such as cyst rupture, which may lead to superinfection, pneumonitis, or anaphylactic reactions due to cyst spillage. Untreated spillage of cyst contents into the pleural space can lead to metastatic infection, which significantly complicates treatment. Cyst-associated symptoms and signs include shortness of breath (SOB), cough, hemoptysis, pneumonia, atelectasis, congestion of the superior vena cava due to mass effect, and peripheral eosinophilia [1].

Diagnosing pulmonary CE can be challenging. The diagnosis is primarily made radiological using computed tomography (CT) scans, which help characterize cysts and identify complications [1]. Serological tests, such as enzyme-linked immunosorbent assay (ELISA), may aid in detecting specific antibodies against *Echinococcus*, but they often have low sensitivity, particularly for extrahepatic cysts [3, 4]. A confirmatory diagnosis often requires histopathological examination of cyst fluid or tissue.

The prevalence and incidence of primary pulmonary CE vary globally, with higher rates in regions where livestock farming is common, such as the Mediterranean, Middle East, Africa, South America, and Central Asia [5]. In endemic areas, incidence rates can reach up to 50 cases per 100,000 population annually. Cases of CE in North America, particularly in Canada, are rare and are primarily linked to Indigenous communities or individuals who have migrated from endemic regions. In Canada, *Echinococcus canadensis* (Genotypes G8 and G10) within the *Echinococcus granulosus sensu lato* complex is considered endemic. Detailed incidence rates remain poorly documented. Between 1940 and 1990, 300 cases were reported among Native Americans in Alaska and Canada, with only three additional cases identified from 1990 to 1999 [6]. Between 2003 and 2024, our center evaluated 131 cases of CE, of which 17 were locally acquired, including cases from Southern Quebec [7, 8].

Treatment options for pulmonary CE require multidisciplinary care and typically include one of the three main strategies, depending on disease extent and stage: surgical removal of the cysts, antiparasitic drug therapy with benzimidazoles, or a watch-and-wait approach. For pulmonary CE, a case-by-case treatment strategy should be applied under the guidance of an experienced physician.

## 2. Case Presentation

A 36-year-old male from the James Bay Cree Territories in Quebec, Canada, with no past medical history, presented to the emergency department (ED) with a two-month history of SOB and cough. Two months prior to the ED presentation, the patient had begun experiencing a dry cough and exertional SOB, along with pleuritic chest pain. Later, the cough became productive with yellowish sputum and was associated with intermittent subjective fever and night sweats. The patient also noted an unintentional weight loss of over 30 pounds in 2 months. The patient initially presented to a local clinic, where he was prescribed amoxicillin for suspected community-acquired pneumonia. After completing a 7-day course of antibiotics, the patient returned to the clinic with persistent symptoms. A chest X-ray revealed a large cavity in the right upper lobe (RUL), prompting his transfer to the McGill University Health Centre in Montreal.

**Figure 1:**
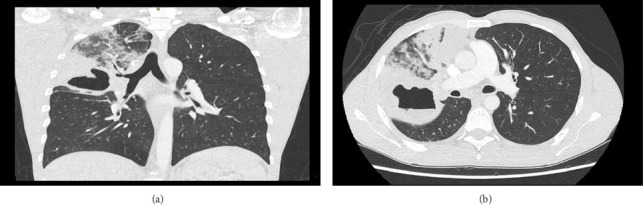
(a) Coronal section and (b) axial section of chest CT scan reveal extensive multifocal consolidation in the right upper lobe, including a large thick-walled cavity in the posterior segment, measuring approximately 6.8 × 7.6 × 6.1 cm. Additional features include clustered and tree-in-bud nodules, along with patchy foci of consolidation in the right middle lobe, lingula, and left lower lobe.

On examination, the patient was febrile with a temperature of 39.1°C; otherwise, his vital signs were within the normal range. Respiratory examination revealed good air entry bilaterally with bronchial breathing in the right mid and upper zones. Laboratory investigations revealed mild leukocytosis (11.57 × 10^9^ cells/L) and a high absolute eosinophil count (1.57 × 10^9^ cells/L) (Table 1). A chest CT showed extensive multifocal consolidation in the RUL with a large thick-walled cavity measuring 6.8 x 7.6 × 6.1 cm in the posterior segment. Additional findings included clustered and tree-in-bud nodules and patchy consolidation in the right middle lobe, lingula, and left lower lobe, a small right pleural effusion, and multistation mediastinal and right hilar lymphadenopathy up to 1.9 cm (Figure 1).

Initially, the patient was started on ceftriaxone and metronidazole for suspected community acquired bacterial pneumonia and/or aspiration. On the second day of admission, three induced sputum specimens were negative for acid-fast bacilli (AFB), and the absolute eosinophil count further rose from 1.57 to 2.00 × 10^9^ cells/L. Upon further history taking, the patient reported that he is a hunter with no dog of his own. Considering the clinical and radiological presentation, the presence of eosinophilia and residence in a region with other confirmed cases in recent years, an echinococcal serology was performed, which came back positive with an optical density (OD) of 1.17 (OD cutoff value, 0.35) (Table 2). The patient was immediately started on albendazole and praziquantel and kept on antibiotics for locally acquired pulmonary CE with suspected cyst rupture and spillage into the pleural and bronchoalveolar spaces, complicated by a superimposed bacterial infection of the RUL.

Thoracic surgeons were consulted for a possible lobectomy; however, given the patient's stability upon admission, the decision was made to treat the patient conservatively with close monitoring. A follow-up chest CT 1 week later showed a decrease in the size of the RUL cavitary lesion to 5.5 × 4.5 cm, with regression of the adjacent consolidation and ground-glass opacities.

**Figure 2:**
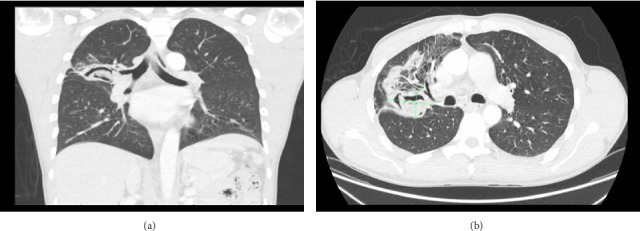
(a) Coronal section and (b) axial section of 1 month follow-up chest CT scan reveals interval decrease in size of the thick-walled cavitary lesion in the right upper lobe which now measures 5.1 × 3.2 cm and interval improvement of consolidative and ground-glass opacities in the right upper and middle lobes.

The patient was discharged on albendazole with therapeutic drug monitoring and follow-up appointments in tropical and geographic medicine, as well as thoracic surgery. One month later, the patient showed significant improvement of his symptoms. A follow-up chest CT revealed a further decrease in the size of the RUL cavitary lesion to 5.1 × 3.2 cm (Figure 2). At the 2-month follow-up, a decision was made to stop albendazole without proceeding to surgical intervention, based on the presumption of a nonviable germinal membrane secondary to bacterial superinfection, the appearance of collapsed cyst membranes on chest CT, and the limited extent of pleural spillage. The patient was scheduled for continued monitoring with a repeat CT chest in 2 months, during which he remained clinically stable with no recurrence of symptoms. A follow-up CT performed two years after initial presentation and 16 months after cessation of antiparasitic therapy demonstrated a significant interval reduction in the size of the RUL posterior segment cavitary lesion, now measuring 2.8 × 1.4 cm in the axial plane, confirming radiologic stability and further resolution.

## 3. Discussion

Diagnosing and managing primary pulmonary CE presents considerable challenges. The clinical manifestations can vary widely, ranging from asymptomatic cysts to large cysts that may rupture and become superinfected with bacteria. Radiological investigations are the primary diagnostic tool for pulmonary CE [1]. Unfortunately, while ultrasound and magnetic resonance imaging are more accurate than CT for the definitive diagnosis of CE, they are seldom possible for lung cysts [1, 9]. This frequently yields diagnostic uncertainty with CT imaging alone. Greater diagnostic confidence can be achieved by combining radiological evidence of a cyst, positive serological tests, and elevated eosinophil counts. However, in cases of unruptured cysts, serological tests often have poor sensitivity, and eosinophilia is usually absent [3, 4, 10].

The management of pulmonary CE is highly individualized, depending on various factors such as cyst characteristics (e.g., size, rupture status, and infection) and whether the disease is isolated to the lung or involves other organs [1]. Surgical removal of a viable cyst remains the primary treatment approach. However, in selected cases—particularly when the cyst is suspected to have already ruptured and become superinfected—medical management with antiparasitic agents may be appropriate. Albendazole is the first-line agent for such cases, due to its efficacy in reducing cyst viability. At our center, praziquantel is added in cases of rupture or spillage to enhance the protoscolicidal effect, based on prior systematic reviews and meta-analyses demonstrating that albendazole–praziquantel combination therapy results in superior scolicidal and anticyst activity, with improved clinical and radiologic outcomes compared to albendazole alone [1, 11]. For asymptomatic, small, inactive CE, a “watch and wait” approach can be applied, with follow-ups ranging from every 6 months to annually. Unlike hepatic CE, percutaneous approaches are contraindicated in pulmonary cysts due to the risk of pleural dissemination [1].

In our case, the differential diagnosis for the patient's presentation was extensive, encompassing bacterial lung abscess, tuberculosis, superinfected congenital cystic adenomatoid malformation, superinfected bronchogenic cyst, and fungal infections, as well as parasitic diseases. A high index of suspicion, along with a detailed patient history regarding habits (e.g., hunting and exposure to dogs and wolves) and geographic origin, is crucial in these cases. The decision for medical management was guided by the presence of bacterial superinfection, radiologic evidence suggesting cyst rupture and membrane collapse, and patient stability. Albendazole therapy was administered and later discontinued after clinical and radiological improvement, with praziquantel given as adjunctive therapy.

Furthermore, the World Health Organization (WHO) classification for cystic echinococcosis is primarily focused on hepatic cysts, with limited description and applicability to pulmonary CE [1, 12], necessitating a case-by-case management approach. This approach should consider factors such as localized versus disseminated disease and whether the cyst has ruptured. In the present case, medical management with albendazole and close follow-up was chosen due to multiple considerations, including the suspicion that the patient's cyst had already ruptured and become superinfected with a bacterial infection. The resolution of symptoms and radiological evidence of cyst size regression indicated successful disease control.

Importantly, long-term monitoring is essential, as recurrence or complications may occur years after apparent clinical resolution. Although our patient demonstrated sustained radiologic improvement and remained asymptomatic 16 months after completing therapy and 2 years after the initial presentation, continued follow-up is warranted. Current recommendations advise monitoring for up to 5 years with periodic imaging to evaluate for recurrence, particularly in nonsurgical cases [9]. Long-term outcomes of medically managed pulmonary CE remain underreported, underscoring the need for additional case documentation and prospective data.

## 4. Conclusion

Pulmonary CE requires individualized management due to its varied presentations. This case highlights both the importance and limitations of imaging and serology in diagnosing pulmonary CE. Albendazole treatment and careful monitoring proved effective, with surgery reserved for complicated or refractory cases. Long-term follow-up and family screening remain critical to detect recurrence and identify asymptomatic cases.

## Figures and Tables

**Table 1:** Initial basic laboratory investigation.

Test	Result	Reference range
White blood cells (WBCs)	11.30	4.50–11.00 ×10^9^ cells/L
Absolute neutrophil count	7.41	1.80–7.70 ×10^9^ cells/L
Absolute eosinophil count	1.57	0.00–0.45 ×10^9^ cells/L
C-reactive protein (CRP)	143.52	0.000–5.00 mg/L
Creatinine	50	55–110 μmol/L
Total bilirubin	13	1.7–18.9 μmol/L
Alanine aminotransferase (ALT)	45	6–45 U/L
Alkaline phosphatase (ALP)	62	53–128 U/L

**Table 2:** Infectious workup and microbiology cultures.

Test	Result	Reference range
Respiratory virus multiplex polymerase chain reaction (PCR)	Negative	Negative/positive
Acid-fast Bacillus (AFB) on sputum	Negative	Negative/positive
Tuberculosis (TB) culture on sputum	Negative	Negative/positive
Blood culture	Negative	Negative/positive
Sputum ova and parasite	Negative	Negative/positive
*Echinococcus granulosus* serology (ELISA)	1.17	< 0.35 negative 0.35–0.44 equivocal 0.45–1.19 low positive > 1.20 high positive

## Data Availability

Materials and data presented in this case study can be obtained from the corresponding author upon request.
